# Mental Health in the Time of the COVID-19 Pandemic: A Scoping Review of Collateral Effects on Common Mental Disorders (CMDs)

**DOI:** 10.3390/ijerph22040478

**Published:** 2025-03-23

**Authors:** Anna Maria Höhn, Leonie Ascone, Luzie Lohse, Dimitrij Kugler, Martin Lambert, Natalia Wege, Felix Wittmann, Steffi Riedel-Heller, Melanie Luppa, Mohamed E. G. Elsayed, René Hurlemann

**Affiliations:** 1 Department of Psychiatry, School of Medicine and Health Science, Carl von Ossietzky University of Oldenburg, Ammerländer Heerstraße 114-118, 26129 Oldenburg, Germany; 2 Department of Psychiatry and Psychotherapy, Working Group Neuronal Plasticity, University Medical Centre Hamburg-Eppendorf (UKE), 20251 Hamburg, Germany; 3 Department of Psychiatry and Psychotherapy, Neuropsychology and Psychotherapy Research Unit, University Medical Centre Hamburg-Eppendorf (UKE), 20251 Hamburg, Germany; 4 Department of Psychiatry and Psychotherapy, Research Group Severe Mental Illness, Early Detection, Integrated Care, University Medical Centre Hamburg-Eppendorf (UKE), 20251 Hamburg, Germany; 5 Institute for General Medicine, University of Düsseldorf, 40225 Düsseldorf, Germany; 6 Institute of Social Medicine, Occupational Health and Public Health (ISAP), Leipzig University, 04109 Leipzig, Germany; 7 Department of Psychiatry and Psychotherapy III, University of Ulm, 89081 Ulm, Germany

**Keywords:** COVID-19, pandemic, collateral effects, common mental disorders, mental health

## Abstract

It is unclear whether the COVID-19 pandemic has had consequences for common mental disorders (CMDs). This scoping review aims to examine direct infection-related (e.g., severe COVID-19 illness), psychosocial (e.g., social isolation), and indirect outcomes (e.g., changes in incidence) that have been particularly discussed so far. A literature search for clinically diagnosed adult CMDs was conducted using Pubmed, Web of Science, and PsycInfo (*n* = 5325). After completion of the screening process, 26 included studies remained for extraction. None of the included studies reported post-pandemic data. The effects appeared to be particularly pronounced for anxiety and obsessive-compulsive disorders in the first year of the pandemic. This was followed by a period of adjustment, during which rates of mental disease and its symptoms largely returned to pre-pandemic levels. Fluctuating rates of CMDs may have had COVID-related causes. Preventive temporary inpatient care could be a protective approach for those at risk or vulnerable, as well as establishing pandemic consultation and building resilience. A gap in the research is the lack of comparisons of CMD data before, during, and after the pandemic to distinguish transient disease rates from chronic disease requiring treatment.

## 1. Introduction

The COVID-19 pandemic left its psychological mark on the population. The restrictive preventive measures, such as lockdowns, and the strictly regulated rules of social behavior, such as social distancing, restricted physical mobility, loss of control, and feelings of insecurity, were often perceived as a burden and characterized the daily life of the population during this period [1,2,3]. The occurrence of increased symptoms such as anxiety and depressed mood in the general population, especially at the beginning of the pandemic, confirms this impression [4,5,6]. In addition, severe cases of COVID-19, requiring intensive medical care or even death, were not only a physical burden but also caused anxiety and psychological distress. All these burdens affected people with mental illness particularly severely [7] and have been systematized into a scheme on which this paper is based [8].

The impact of the COVID-19 pandemic on mental health has increasingly become a subject of scientific inquiry. A recent framework differentiates the sequelae of the pandemic into three categories: psychosocial effects (PSEs), indirect total effects (ITEs), and direct total effects (DTEs). These categories are organized in a matrix scheme [8]. This encompasses psychosocial collateral effects, emerging comorbidities, and physical aspects. Initial analyses have indicated that the politically initiated measures to protect the population, such as lockdowns and social distancing, have contributed to the objective of reducing the risk of infection. However, a consequence that was not considered during the acute phase of the pandemic was that restrictions on individuals and social activities resulted in disadvantages (e.g., financial insecurity and loss of social participation). These disadvantages were particularly prevalent among vulnerable groups, including individuals with genetic and/or psychosocial predispositions and/or a pre-existing disorder. These individuals were not only at an elevated risk of infection with the virus and its associated consequences, but they were also more susceptible to the exacerbation and progression of pre-existing medical conditions [9]. Consequently, they have exhibited a greater degree of distress compared to the general population. For example, individuals with specific vulnerabilities, such as chronic pre-existing conditions, were increasingly advised to adhere to social distancing measures, which subsequently resulted in elevated stress levels [10]. The theoretical framework and methodological proposal encouraged the simultaneous consideration of the potential effects of the virus and the psychosocial consequences of the pandemic’s dynamic and political containment measures [8]. This included the estimation of total indirect consequences on mental health in a systematic manner. The objective and inquiries of the authors proposal were to enhance monitoring and, consequently, the evaluation of whether pertinent psychological effects may exceed infection-related effects or vice versa. An evaluation system for future pandemics could be established by estimating the relative importance of preventive measures in maintaining the physical, social, and psychological integrity of the population.

In order to further develop these proposals for evaluating and assessing the consequences of prevention measures for future pandemics, this review aims to provide a broad international overview of the literature [11]. In the preceding search for an overview of the existing literature on mental disorders in the population, it was noted that the spectrum of anxiety disorders includes some of the most common mental disorders and has received a lot of scientific attention, as shown in Section 1. Consequently, the study search for this scoping review was expected to focus on a large number of anxiety disorders. It is evident that a multitude of other mental illnesses are associated with CMDs. In the following chapter (Section 2), the further relevant disorders for this review are explained and defined under CMDs. Partly due to massive prevention-related restrictions, it was anticipated that a considerable number of primary studies had been conducted with the initial assessment of patients. However, there is a paucity of studies employing clinical diagnosis, such as the categorization of these specific mental effects in adults with CMDs during the pandemic in comparison to other population groups.

This study is distinguished from previous research in that it provides a systematic approach to understanding the various effect dimensions associated with CMDs. This study thus contributes to a larger, potentially affected population group and, by including only clinical diagnoses, improves the assessment of actual consequences that result in some form of therapeutic need. Accordingly, the objective of this review is to facilitate the monitoring of the impact of the pandemic on public health, in accordance with the recommendations set forth by the World Health Organization (WHO) [12]. The recommendations resulting from this research may inform the development of a national mental health surveillance system in Germany [13]. This knowledge could facilitate the provision of evidence-based counsel regarding measures to ensure the physical and mental health of vulnerable groups and the general population.

The following research questions are addressed in the present review:

1. Have the COVID-19 pandemic and its consequences and restrictions generated indirect total effects (ITEs), such as increases in the incidence and prevalence of common mental disorders (CMDs) in the general population, the progression of pre-existing CMDs, or the development of mental comorbidities in adults with pre-existing CMDs?
2. Which direct COVID-19-related total effects (DTEs; e.g., contagion rates, severe COVID-19 illness with ICU admission, and COVID-19-related deaths) and psychosocial factors (PSEs; e.g., loss of occupation and lack of access to healthcare facilities) have been investigated and recorded in the identified studies of adults with pre-existing CMDs?

## 2. Materials and Methods

This study was conducted in accordance with the PRISMA Extension for Scoping Reviews (PRISMA-ScR) checklist [14]. 

The a priori protocol was finally registered on 12 April 2024 and comprises the PECOS scheme with the inclusion and exclusion criteria and the definition of CMDs, with the corresponding ICD-10 codes (https://osf.io/wxdyq/ accessed on 12 April 2024). Furthermore, the document provides a comprehensive rationale for the selection and definition of CMDs, taking into account their 12-month prevalence, their public health relevance, and the necessity to avoid overlap with the research conducted by the collaborating group, which investigates the effects of severe mental illnesses (SMIs) (https://osf.io/9eyd5/ accessed on 12 April 2024).

In the initial subchapter (see Section 2.1), the included definition and associated disorders falling within the category of CMDs, along with the various effect dimensions, are described and defined. The second subchapter (see Section 2.2) outlines the methodology employed for the identification of pertinent studies, utilizing a search string. The third subchapter (see Section 2.3) elucidates the selection process of studies in accordance with inclusion and exclusion criteria, resulting in the formulation of a screening strategy. The fourth subchapter (see Section 2.4) presents the results in a systematic manner. The outcomes gathered from the various sources are then transferred to the matrix scheme. The results are presented in a narrative format using an evidence mapping approach. This is done to illustrate which components of the matrix have been more or less researched, identify patterns of research activity, and summarize related findings on direct virus-related and indirect psychosocial effects, as well as indirect effects related to mental illness. The proposed matrix scheme, which includes the specific vulnerability and risk factors, can be found in the Supplementary Materials. The aforementioned approach was oriented towards the methodological procedure of scoping reviews [14,15,16].

### 2.1. Definitions

#### 2.1.1. Common Mental Disorders (CMDs)

The term common mental disorders is used to describe a group of frequently occurring mental illnesses that have a high prevalence in the general population. Such disorders include, for example, anxiety disorders, affective disorders, obsessive-compulsive disorders (OCDs), and stress reactions [17,18]. It is also pertinent to mention the syndrome of burnout in this context. Although not classified as a disease, it describes difficulties with lifestyle management. With an underestimated prevalence of 3%, it has increasing public health relevance and is therefore also considered [19,20]. To avoid any potential overlap with the collaborating research team (https://osf.io/9eyd5/ accessed on 21 March 2024), all forms of depression, schizophrenia, psychosis, and bipolar disorder have been excluded from the present study. Another rationale for the exclusion of these diagnoses is that numerous studies do not provide precise data regarding the severity of conditions such as depression. Instead, they employ a superordinate coding system. As a result, a more precise differentiation has been challenging to achieve. To prevent an exhaustive investigation of numerous disorders, other potentially relevant conditions, such as personality disorders and eating disorders, were excluded from the search.

The precise list of included disorders according to the International Classification of Diseases, 10th Revision (ICD-10) and their definition according to prevalence, public health relevance, and differentiation to severe mental illness (SMI) have been specified in the a priori protocol (https://osf.io/wxdyq/ accessed on 12 April 2024) and are listed in the Supplementary Materials hereafter with diagnostic coding F40 to F.43 and Z73. The following disorders were included: phobic anxiety disorders, panic disorder, generalized anxiety disorder, obsessive-compulsive disorder, acute stress reaction, post-traumatic stress disorder, adjustment disorder, and burnout.

#### 2.1.2. Psychosocial Effects (PSEs)

As previously stated, psychosocial stress interacts with pre-existing vulnerability. It is anticipated that the ten effects enumerated below may have contributed to a deterioration in mental health outcomes among vulnerable populations, particularly individuals with CMDs. Ascone et al. [8] delineated ten dimensions of psychosocial effects that may have occurred during the course of the pandemic and that may interact with vulnerability to influence an individual’s mental health status. Social contacts and relationship factors (PSE1) and coping strategies (PSE2), as well as physiological health status (PSE3) and changes in lifestyle (PSE4), may have been affected in a positive or negative manner by the pandemic. The occurrence of trauma or adverse experiences (PSE5), fluctuations in work-related stress (PSE6), and environmental stress (PSE7) may have undergone changes during the pandemic, potentially providing relief or imposing additional burdens. Demographic factors (PSE8), such as an increasing educational gap, may be associated with a higher vulnerability towards the development of mental disorders [20]. In addition, a lower income is associated with an increased risk of mental disorders, whereas a higher income is associated with a decreased risk [21]. Moreover, structural factors (PSE9) are of paramount importance with regard to the administration of medical treatment. As an illustration, the implementation of lockdowns has considerably impeded access to medical facilities. Furthermore, overall quality of life (PSE10) may have been impacted by the pandemic and associated regulations, which is why this dimension has been included in the current review.

#### 2.1.3. Indirect Total Effects (ITEs)

In the preceding work, the term indirect total effects was used to describe the comprehensive impact of the pandemic (and the subsequent psychosocial effects) on mental health [8]. For example, ITEs may be associated with an elevated prevalence or incidence of mental disorders [21]. Additionally, other outcomes may befall the patient within this category, including a deterioration of the prognosis, a relapse, a period of remission, a recurrence of symptoms, suicide, an increase in acute or coercive hospitalizations due to mental disorders, an increase in coercive measures when hospitalized, or the emergence of mental comorbidities. For example, a case-control study conducted in China in 2020 demonstrated an increase in the prevalence of anxiety, depression, stress, sleep disorders, and suicidal ideation among psychiatric patients. Additionally, the emergence of comorbid post-traumatic stress disorder (PTSD) was observed in approximately one-third of the patients [22].

#### 2.1.4. Direct Total Effects (DTEs)

During the pandemic, the primary focus was on concerns regarding potential infection with emerging variants of the coronavirus, which were associated with varying symptoms, symptom severities, and virulence. These correlations were linked to the duration and frequency of exposure. The risk of a severe case necessitating hospitalization, additional oxygen requirements, intubation and ventilation, or, in the most severe cases, death was primarily associated with specific characteristics and pre-existing illnesses. These include advanced age, obesity, hematological neoplasms, metabolic disorders such as diabetes, and cardiovascular, pulmonary, and renal diseases, as well as dementia, mental disabilities, and mental illnesses [23]. Nevertheless, it has been demonstrated that pharmacological interactions with other pharmaceutical agents, including antipsychotics, antidepressants, and anxiolytics, can impact the efficacy of antiviral treatments for SARS-CoV-2. Therefore, it is essential to carefully weigh the potential benefits and risks associated with such interactions before initiating treatment [24]. In addition to the acute health risks previously outlined, the long-term consequences of infection are becoming increasingly relevant. These are classified as post-COVID syndrome versus long-COVID syndrome.

In conclusion, it is possible that individual or multiple PSEs, ITEs, and DTEs may interact with each other. For example, social and relationship-related stressors resulting from quarantine measures have been identified as a potential risk factor for the development of mental disorders during the pandemic. Conversely, the presence of a mental illness and an unhealthy lifestyle, for example, can also increase the risk of infection.

### 2.2. Study Searches

The databases PubMed, Web of Science, and PsycInfo were utilized due to their comprehensive scope and relevance to medical and psychiatric topics. The study search in the three databases was conducted on 9 April 2024. It then took 15 weeks to complete the two screening rounds and to include the studies that appeared relevant for this review.

#### Search Strings

The terms used to describe the COVID-19 pandemic, as well as the specific diseases associated with it, were identified as keywords for the title and abstract. These included the inclusion of adults and the exclusion of studies on children and adolescents. The full list of keywords can be found in Table 1.

### 2.3. Study Selection

The PECOS framework was used for the structured formulation of a scientific research question, including the population, exposures, comparators, outcomes, and study types [25]. Each study’s population consisted of individuals aged 18 years and above who met the criteria for one or more of the aforementioned CMDs based on the ICD-10. The focus was on adults, as children and adolescents have different needs and may experience different effects in the development of mental disorders. It is imperative to consider the impact of the pandemic on this target group in a separate analysis. The data collection period was required to encompass at least one point in time during the course of the pandemic dated from 11 March 2020 until 5 May 2023, and it was imperative that no overlap occur with other potential sources of exposure, such as natural disasters. The outcomes were defined as the PSEs 1-10, ITEs, and DTEs. It was also necessary for each study to include a comparison group in form of a comparison to other mental illnesses and/or the healthy population. We considered longitudinal, cross-sectional, retrospective, and observational studies. The following types of publications were excluded: preprints, protocols, intervention studies, and theoretical publications. All relevant data can be found in Table 2.

**Figure 1:**
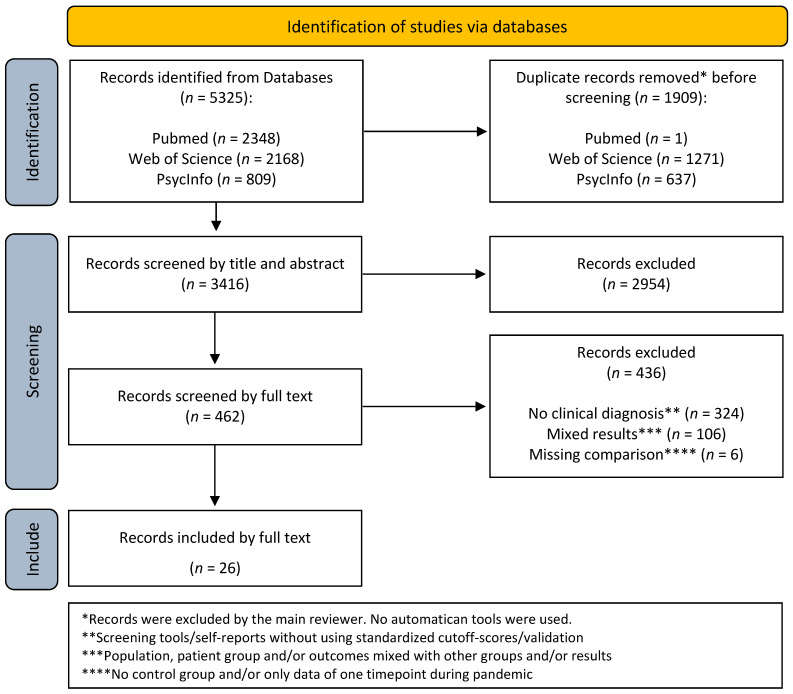
PRISMA flowchart of the screening process.

The list of hits was stored using the literature management program Zotero 7.0.8. This included, at a minimum, the year of publication, author(s), title, abstract, and Digital Object Identifier (DOI) for the initial screening round. Additionally, the full text was included for the second screening round. Manual removal of duplicates was conducted. The complete screening process is illustrated in a PRISMA flow diagram (Figure 1). Of the 5325 initial hits identified across the three databases, 3416 were subjected to screening after deduplication. A total of 2954 hits were excluded following a review of the title and abstract. Of the 462 full texts reviewed, 436 were excluded due to the absence of a clinical diagnosis, the presence of mixed results with other disorders, and/or the absence of a comparison group. Following the second screening process, 26 hits were deemed suitable for inclusion in the review.

In the initial screening phase, the titles and abstracts of the identified studies were evaluated according to the established inclusion and exclusion criteria (https://osf.io/wxdyq/ accessed on 12 April 2024). The selection of abstracts for the first screening round was conducted by one reviewer. The objective was to identify primary studies that were conducted during or prior to the pandemic in adults with and/or without CMDs, with and/or without control groups, and examined mental health conditions (PSEs, ITEs, and DTEs) during the pandemic based on the information available in their titles and abstracts. If the review indicated that a source was pertinent, it was then sorted into the corresponding subfolder in the literature management program. In order to facilitate discussion and provide access to further primary studies, relevant reviews and meta-analyses were saved. In the second screening round, the full texts of the remaining hits were evaluated to ascertain whether they included suitable outcomes on the PSE, ITE, and DTE dimensions. This was done in accordance with the previously established inclusion and exclusion criteria. In cases where a categorization of a single study appeared unclear despite orientation towards the protocol requirements, the matter was discussed within the working group (AH, LA, LL, and DK).

It was anticipated that a smaller number of studies would be identified that had made a clinical diagnosis of the specified CMD, as opposed to a much larger pool of studies that had applied self-report or screening tool-based definitions of CMDs. Nevertheless, it is acknowledged that the exclusive utilization of self-assessments is susceptible to a self-report bias, particularly in the context of mental health [26]. It is therefore recommended that a self-assessment questionnaire should only be used for diagnosis in combination with further information and the examiner’s assessment during a personal interview. To provide a qualitative answer to the key question of the change in mental health during the pandemic and to maintain the study sample at an appropriate size, only diagnostic interviews or a diagnosis according to the ICD-10 were considered, as specified in the a priori protocol. Furthermore, a self-reported diagnosis was permitted, provided that the study participant clearly identified the disorder in question and indicated that the diagnosis had been rendered by a qualified healthcare professional. In order to assess changes in the symptoms or symptom severity of a clinically diagnosed CMD during the pandemic (which constituted part of the ITEs), or to record other relevant factors (PSEs and DTEs), self-assessment was accepted.

### 2.4. Evidence Synthesis and Reporting

The results are presented in accordance with the specifications set forth in the protocol, accessible via the following link: https://osf.io/wxdyq/ accessed on 12 April 2024. The PSEs from 1–10, ITEs, and DTEs, which were referenced in Section 2.1, are presented in Section 3 as individual subsections, including Section 3.1, Section 3.2, Section 3.2.1, and so on. An evidence map (Table 3) provides a graphical representation of the number of included hits and the PSE, ITE, and DTE outcome dimensions. This allows for a graphical overview of which dimensions have been studied.

## 3. Results

### 3.1. Study Pool

The majority of the included studies were conducted in Europe (*n* = 13) and North and South America (*n* = 11). One study was conducted in Asia and one was conducted in Australia. The number of data points included in the study sample varied. As anticipated, no uniform definition of the overarching term CMD was identified across the included studies. Only a limited number of authors provided justification for the selected CMDs as common, indicating that they were selected due to their high prevalence [27,28]. The majority of studies (25 of 26) concentrated on anxiety spectrum disorders, encompassing generalized anxiety disorder (GAD), phobias, panic disorder (PD), and/or neurosis as obsessive-compulsive disorder (OCD). The literature on stress reactions is relatively limited (six of twenty-six studies) and no studies were identified that met the criteria for inclusion in the burnout category. Among the phobias, social anxiety disorder (SAD) and agoraphobia were subjected to analysis [29,30,31,32,33]. Additionally, some studies have employed a comparative approach, examining the relationship between SMIs and CMDs, or alternatively, examining SMIs as part of a larger research project [33,34,35]. Only three studies controlled for infection status [36,37,38].

### 3.2. Evidence Map

A greater number of studies employ a retrospective approach (eleven studies classified as types E and F) than a cross-sectional approach (eight studies classified as types C and D) or a longitudinal approach (seven studies classified as types A and B). A detailed classification of the various study types, from A to F, can be found under the evidence map (Table 3). The majority of studies (25 out of 26) examined at least changes in incidence/prevalence and/or emergency department (ED) visits and/or symptom worsening in pre-existing diseases and are summarized under ITEs. The evidence base for PSEs and DTEs is limited. The clinical diagnoses of the included studies were conducted through a variety of methods, including the use of ICD-10 coding by healthcare professionals, ICPC (International Classification of Primary Care)-2 codes in primary care settings, web-based interviews, telephone-based or in-person interviews (CIDI 3.0 and M.I.N.I.) with healthcare professionals, and data from insurance records, hospitals, or primary care settings. The complete set of 26 included studies, accompanied by detailed results, can be found in the Supplementary Materials. The evidence map (Table 3) presents the number and distribution of the investigated effects in CMDs and the list of the different study designs.

#### 3.2.1. PSE1—Social Effects

Of the studies included in this review, only two assessed PSE1. The impact of OCD on social functioning was more pronounced than that observed in individuals without OCD [36]. However, positive changes in terms of increased contact with family members during the pandemic have also been reported by individuals with OCD and a healthy control group [39].

#### 3.2.2. PSE2—Coping and Emotional (Self-)Regulation

Four of the included studies assessed PSE2. In the domain of psychological status and coping strategies, the identified studies addressed a range of issues, including fear of contamination and danger, xenophobia, and the use of controlling and calming measures [29]. In the case of PD, SAD, and GAD, these tended to decrease over time, whereas the controlling and calming measures remained in OCD. The prevalence of anxious symptoms in individuals with OCD was approximately five times higher than in healthy controls [36]. Patients with OCD have been found to exhibit lower resilience than healthy controls. However, evidence suggests that higher resilience, both in individuals with OCD and in the control group, is associated with more stable symptoms, and vice versa [39]. In 2021, a stronger tendency to avoid situations was observed in individuals with SAD, while the primary and secondary coping strategies employed by this group were found to be similar to those observed in the healthy control group [31].

#### 3.2.3. PSE3—Physiological Health Status

Only one of the included studies assessed PSE3. The study revealed a significant correlation between anxiety disorders and an increased prevalence of COPD and asthma among participants [40].

#### 3.2.4. PSE4—Lifestyle Changes

No evidence for this psychosocial effect dimension was identified.

#### 3.2.5. PSE5—Traumatic Experiences

A mere three of the studies included in the review assessed PSE5. In the domain of traumatic experiences in the form of traumatic stress symptoms, patients with PD, GAD, OCD, and PTSD exhibited elevated symptoms at the onset of the pandemic which subsequently diminished over time [29]. Stress symptoms were reported to be approximately twice as high in individuals with SAD compared to healthy controls [31]. Individuals diagnosed with OCD reported a greater number of lost loved ones due to the impact of the COVID-19 pandemic than a healthy control group [36].

#### 3.2.6. PSE6—Job-Related Effects

Of the studies included in the review, only two assessed PSE6. In the domain of the impact of the COVID-19 pandemic on work, as manifested in increased stress at work and school, between 36% and 53% of participants with a diagnosis of psychiatric disorder, including PTSD, GAD, OCD, and SAD, reported feeling more burdened. In comparison, only 21% of the healthy control group reported impaired functioning due to the ongoing impact of the pandemic at the conclusion of the third wave [29]. The impact of OCD on occupational functioning was more pronounced than in individuals without the disorder [36].

#### 3.2.7. PSE7—Environmental Exposures

No evidence for this psychosocial effect dimension was identified.

#### 3.2.8. PSE8—Socioeconomic Effects

Of the studies included in the review, only two assessed PSE8. The fear of potential socio-economic consequences was initially high at the onset of the pandemic, but subsequently declined in individuals with PD, SAD, GAD, OCD, and PTSD until 2021. Moreover, the same study revealed that the incidence of self-harm associated with anxiety disorders had declined markedly in impoverished regions [32].

#### 3.2.9. PSE9—Structural Effects

A mere three of the studies included in the review assessed PSE9. In the domain of impact on reduced participation and reduced daily activities due to structural hurdles, individuals with PD, SAD, GAD, OCD, and PTSD reported experiencing greater restriction than healthy controls [29]. The frequency of referrals to medical facilities for the treatment of anxiety disorders declined during the pandemic relative to the period preceding it [32]. The impairment of social life through difficult structural access was a more prevalent phenomenon in individuals with OCD than in healthy individuals [36].

#### 3.2.10. PSE10—Quality of Life

A single study included in the review assessed PSE10. A majority of the sample of individuals with OCD reported a decline in their quality of life at the outset of the pandemic, with three-quarters of respondents indicating at least a slight deterioration in their quality of life in spring 2020 [41]. The results of this study should be interpreted with caution, as only one study was identified and the study’s sample size was relatively small, comprising only 201 participants.

#### 3.2.11. ITE

The majority of the 25 studies included in the analysis assessed different ITEs. For purposes of clarity, the disease rates, ED visits, and symptom changes are presented separately for the ITE outcome category. In the absence of pre-pandemic comparative rates, for instance for 2020, these are not available or do not emerge from the study. In this instance, only comparative rates for multiple survey dates within the specified pandemic periods are available.

#### 3.2.12. Emergency Department (ED) Visits

Five of the included studies assessed ED visits. ED visits are based on clinical diagnosis, yet they do not provide information about specific disease rates. However, they can be interpreted as an indication of how acutely the need for medical treatment may be changing and also of changes in healthcare provision. For example, a lack of care due to reduced low-barrier services may result in more severe disease progression, as well as improved access to facilities. The number of visits for anxiety disorders, OCD, and stress-related disorders (SRDs) remained stable overall before and after the delta variant (April to August 2021) and before and after case peaks (December 2020 to February 2021). However, a negative peak was observed in all disorders during April/May in the USA, a period which also exhibited lower rates than in 2018 [42]. The current period is marked by a significant increase in the number of business closures and a twofold rise in mortality rates associated with the pandemic in the United States [43]. In comparison to the pre-pandemic period, visits to facilities other than emergency departments for anxiety disorders and substance-related disorders remained stable [44]. In the context of anxiety disorders, Italy, which was particularly affected by the direct consequences of the pandemic, observed a reduction in the number of visits of up to 30% in 2020 compared to 2019, prior to the pandemic [45]. In Australia, there was an increase in emergency department visits for anxiety disorders from 15 to 20% during the initial nine weeks of the lockdown, particularly among individuals aged 26-45 years. This trend then reversed, with a subsequent decline in visits [34].

#### 3.2.13. Disease Rates (Incidence and Prevalence)

Eleven of the included studies assessed disease rates. The majority of identified studies focused on analyzing the incidence rates of anxiety disorders. This is likely attributable to the fact that anxiety disorders are among the most common disorders. In Germany, the number of incident cases of anxiety disorders initially increased by 40% and then exhibited a decline [40]. Spain shows similar data [46]. The incidence of anxiety disorders and OCD has not increased significantly in the United States [38]. A modest increase was observed in the prevalence of self-reported clinical diagnoses of PD and SAD in the USA during the initial year of the pandemic [29]. The incidence of anxiety disorders initially demonstrated a decline at the onset of the pandemic in England, followed by an increase after the initial wave [32]. The prevalence of anxiety disorders in the Netherlands has remained relatively stable [28]. In the Czech Republic, the prevalence of PD remained stable, while the prevalence of social phobia doubled, reaching 4%. Similarly, GAD doubled, reaching 6%. The prevalence of PTSD increased from 1% to 3%, while the prevalence of agoraphobia increased from 5% to 9% [33] and overall anxiety disorders increased from 8 to 13% by the end of 2020 [47,48]. There has been a notable increase in the prevalence of adjustment disorder in Serbia, rising from 2% in 2018 to 11% in 2020 [35]. The prevalence of anxiety disorders in Norway remained low at 5%, and in Sweden at 4%, throughout the course of the pandemic [49].

#### 3.2.14. Changes in Psychopathological Status in Individuals with Pre-Existing CMD

Thirteen of the included studies assessed changes in pre-existing CMD. A worsening of symptoms has been reported for pre-existing anxiety disorders, including panic disorder (PD), generalized anxiety disorder (GAD), social anxiety disorder (SAD), as well as obsessive-compulsive disorder (OCD) and post-traumatic stress disorder (PTSD) at the onset of the pandemic, with a notable surge in incidence. This trend was reversed after the introduction of vaccinations until the conclusion of the third wave in 2021 [29,30]. In individuals diagnosed with SAD, the prevalence of avoidance behaviors and anxiety symptoms was found to be twice as high as in a control group of healthy individuals in 2021 [31]. The incidence of self-harm in individuals with anxiety disorders remained consistent with pre-pandemic levels. However, during the initial phase of the pandemic, the prescription of benzodiazepines was notably elevated in the elderly (aged 80 and above) cohort, before subsequently stabilizing [32]. A comparative analysis of data from the pre- and post-pandemic periods revealed a decline in the prescription of psychotropic medications for the treatment of anxiety disorders [40,50]. In 2021, OCD symptoms were approximately twice as high as in a control group. Furthermore, the majority of respondents indicated that their symptoms had worsened since the onset of the pandemic [36,39,41,51], especially among patients who reported having their diagnosis for 10 years or less [52]. A third of patients indicated that they had experienced an improvement in their symptoms due to the increased social acceptance of strict hygiene measures during the pandemic. Furthermore, respondents indicated that there was a reduced risk of contamination due to social distancing and an increase in time spent at home [41]. The prevalence of suicide risk in individuals with anxiety disorders and PTSD in the Czech Republic reached 21% by the end of 2020, a significant increase from the 4–6% observed in the general population [33]. Among patients with adjustment disorder, 32% reported suicide attempts after the first lockdown in 2020 [35].

#### 3.2.15. Mental Comorbidities

Only one of the included studies assessed new mental comorbidities. In a single prospective cohort study with healthy controls, it was reported that a patient with OCD developed a PD during the pandemic [39]. Otherwise, either no comorbidities had been measured across the examined studies or they were not reported.

#### 3.2.16. DTE

A mere three of the studies included in the review assessed DTEs. Individuals with anxiety disorders exhibited a significantly higher prevalence of pulmonary diseases, including asthma and chronic obstructive pulmonary disease (COPD), compared to the general population. Consequently, they demonstrated an elevated risk of developing severe forms of COVID-19 [40]. On the other hand the risk of developing an anxiety disorder in the event of severe COVID-19 is increased by 10–30% [38]. The mortality risk associated with SARS-CoV-2 infection was not elevated among individuals with anxiety disorders compared to healthy controls in New York City [37]. A greater proportion of individuals with OCD who contracted the SARS-CoV-2 virus required hospitalization and exhibited more severe symptoms than those in the healthy control group [36].

## 4. Discussion

The objective of the review was to provide an overview of the international evidence on the psychosocial, direct, and indirect effects of the COVID-19 pandemic on individuals with a CMD. The aim was to investigate whether and to what extent the restrictions, measures, and consequences of the virus itself have led to a change in new and pre-existing CMDs. Finally, 26 studies with clinical diagnoses were included in this review.

### Analysis of the Study Results

Disease rates, symptom progressions, and PSEs in CMDs, especially in anxiety disorders and OCD, increased significantly at the beginning of the pandemic in most of the studies [29,30,31,39,40,41,46], before declining as the pandemic progressed. The declining figures may signify a form of adaptation response, whereby those affected initially experience a sense of overwhelm in the face of the pandemic situation. However, with time, they tend to adjust to the new reality and hygiene measures, albeit at a somewhat slower pace than the healthy rest of the population [29,39]. This could be the first sign that no treatment may have been required in the long term or that the telemedical care provided was adequate [45]. It is important to note that reactions in the form of worsening symptoms should not be immediately and universally classified as pathological. The preceding paragraph has demonstrated that there was an increased prevalence of CMDs, particularly during the initial phase of the pandemic. However, this trend stabilized after the initial period of uncertainty, which included isolation, lockdowns, and fluctuations in case numbers. Fluctuating values could appear to be a natural adaptation process in which uncertain CMD patients must first find their way back into the new social order, a process that may require more time than that of the healthy population. In particular, this necessitates the investment of time and the formulation of strategies in advance to mitigate these effects in the event of future pandemics. In periods of high demand on emergency departments and an insufficient healthcare system, it is crucial for individuals with CMDs to develop self-management skills and utilize evidence-based strategies for stabilization, which can be practiced with their healthcare professionals outside of the pandemic context. This also applies to susceptible individuals in the general population. Resilience could be an essential component for stabilization [39] and should be focused on in the future.

The majority of studies have analyzed surveys or health data at one or more points in time prior to the onset of the pandemic and at the beginning of the pandemic, particularly during the initial year. This allows for an overview of the initial effects of the pandemic, which naturally suggests higher negative effects. These include a 40% higher risk of being diagnosed with an anxiety disorder in the first six months of the pandemic [42]—but it does not allow a long-term comparison. It is notable that none of the 26 studies included in this review provide long-term comparisons with post-pandemic data. This is a crucial omission, as such comparisons are essential for estimating trends. In the initial stages of the pandemic, which were marked by social unrest, the provision of precautionary inpatient treatment for patients with OCD or the availability of pandemic consultations could have a beneficial impact. The reduction in the incidence of anxiety disorders in hospital settings may be indicative of a protective effect, as those affected are not exposed to the often-changing social and societal dynamics (such as panic and hoarding behaviors) that can occur in the community [50]. Instead, they are in a protective environment.

It is also possible that these effects are caused by a SARS-CoV-2 infection, rather than solely as a consequence of social distancing and restrictive measures. Only three studies controlled for infection status [36,37,38], which may be deemed problematic since there is increasing evidence of associations between severe COVID-19 cases and the development or aggravation of mental disorders [53] and between long-COVID and the development of mental disorders [54,55,56]. For example, there may be an elevated risk of developing an anxiety disorder following a severe case of COVID-19. This is postulated by the authors of the study to be due to an adverse combination of biological inflammatory processes, hypoxia, and traumatic events in the clinical setting [38].

Furthermore, it is notable that the results for pre-existing CMDs exhibit a high degree of consistency across countries and regions. In Eastern Europe, for instance, Serbia and the Czech Republic have reported an increase in new cases and a worsening of symptoms associated with CMDs [33,35,47,48] than in the US [29,42,43]. This may be attributable to the lower acceptance of mental illness and the concomitant under-provision of psychiatric facilities and services in this region. The authors of the study in Norway and Sweden posit that the altered statistics of reduced medication usage are most likely attributable to disparate healthcare provision [49]. In this regard, the disparate country-specific prevention measures and infection numbers introduce an element of uncertainty in the interpretation of the results. These two factors (preventive measures, e.g., lockdowns, as well as infection numbers) could be subjected to further analysis in conjunction with disease rates in order to ascertain any changes in CMDs in relation to them.

## 5. Strengths and Limitations

One of this review’s key strengths is its focus on studies that either included a suitable comparison group or at least two points in time. This approach allows for the comparison and assessment of effects on the healthy population in relation to the potential increased burden on CMD patients. Moreover, only studies in which pre-existing or new clinical diagnoses of CMD were present were included. This enhances the relevance of the findings and differentiates the methodology from other studies that did not impose this criterion with such rigor. Therefore, the investigation of this topic is not only highly relevant for the management of pandemic stress, but should also inform the prevention of future errors in the management of social crisis situations.

There are some limitations that need to be noted. A total of 11 out of the 26 studies included a retrospective assessment of the pandemic situation at various points in time. This could have resulted in a distortion of the respondents’ perceptions, which could in turn have influenced the results in both positive and negative ways. Moreover, it is important to note that prevalence studies and incidence studies are not synonymous. Prevalence studies provide an estimation of the prevalence of a disease at a given point in time, whereas incidence studies offer insights into the actual number of new cases and disease dynamics. Additionally, alterations in ED visits merely reflect a shift in the population’s inclination to seek medical assistance and, consequently, may indicate a change in the overall level of distress within the community. These limitations are not based on a methodologically deficient approach, but rather reflect the diversity of studies at this point in the literature review. These restrictions are mitigated by the exclusive use of diagnosed CMDs, which can indicate a true disease value. A further limitation is that only one reviewer was primarily responsible for the review process, which means that there is no interrater reliability.

The initial identification of studies yielded a considerable number (*n* = 5325) of hits, yet only a modest number (*n* = 26) were ultimately included in the screening process. This can be attributed to the fact that a considerable number of incidence and prevalence studies employed self-report assessments, in some instances solely as online surveys, as a consequence of the implementation of restrictive preventive measures or because face-to-face interviews could only be conducted in accordance with rigorous hygiene protocols. The critical distinction lies in whether the assessment identifies solely the symptoms of CMD or whether a diagnosis is confirmed by medical professionals when the affected individual seeks treatment at a medical facility. Nevertheless, the use of self-reporting is a standard practice in public health research. However, this approach was not employed in the present review to enhance the potential validity of diagnoses and to maintain the study sample’s manageability. Secondly, the reason for the limited number of included studies may be attributed to the inconsistent definition of the term CMDs and the resulting variety of mental disorders that are counted among them. In the included studies, the term CMDs was only used by seven authors [27,28,43,45,46,47,48] and was interpreted by each of the seven authors with different disorders. Anxiety disorders and depression were the most frequently diagnosed conditions classified as CMDs. Further research is required to enable a more precise evaluation. It would also be beneficial to establish a unified terminology for CMDs that encompasses the five most prevalent disorders, thereby facilitating more coherent and effective clinical practice.

With regard to relevance and the assessment of external validity, two further points should be noted as limitations. The very different study sizes, the different types of data (study participants or health data from health systems), and the countries in which each study was conducted cannot be generalized. As previously discussed, countries were subject to different prevention measures and case numbers. In addition, no generalizable conclusions can be extracted from study sizes of <100 participants. A small-scale investigation of country-specific differences with comparably large study sizes is required here.

### Suggestions for Politics and Clinicians and Recommendations for Additional Research

The number of cases and the severity of symptoms associated with existing CMDs appear to have increased significantly at the onset of the pandemic. However, these trends have since stabilized in many studies following an initial adaptation period. In contrast, patients with pre-existing OCD appear to be the most vulnerable, reporting the highest levels of distress during the pandemic. It may be beneficial to provide protection for individuals at high risk or high vulnerability to CMDs from social panic and hoarding at an early stage in a temporary inpatient facility. This could include individuals who have achieved a high score in the vulnerability–risk matrix. A preventive inpatient stay could serve to mitigate the initial acute phase of a comparable pandemic by removing patients from the uncertain environment of a society that is undergoing daily changes. In an inpatient setting, there are not only contact persons directly on site, but there is also the possibility of personal retreat and crisis intervention, which are both beneficial in such circumstances. The implementation of legally prescribed measures can be conducted under the guidance of a medical professional and integrated into the patient’s treatment plan. It may also be beneficial to provide reassurance through telemedical counseling or a pandemic consultation. Those with CMDs should also be empowered by fostering resilience through the practice of strategies learned in advance for future pandemic events.

The investigation of stress-related disorders and burnout, as well as the psychosocial effects associated with all CMDs, has been less comprehensive. The duration of disease is also a relevant factor which is rarely investigated in cases of pre-existing CMD. It is therefore important to differentiate between temporary and chronic CMDs in order to assess the long-term impact on the healthcare system and the pandemic as the cause of genesis. In order to establish comparability, it might also be useful to subdivide by sample size, number of COVID-19 cases, and country-specific measures. Furthermore, a more detailed breakdown of the PSEs, ITEs, and DTEs could be considered in order to facilitate more precise identification of research priorities and gaps.

## 6. Conclusions

In conclusion, in addition to the impact of the global COVID-19 pandemic, there has been a notable shift in societal attitudes towards mental health, leading to an increase in the number of individuals seeking and receiving diagnoses. The early phase of the pandemic had an especially significant impact on individuals with CMDs, particularly in the context of anxiety disorders and OCD. Additionally, disease rates increased at the outset of the pandemic in the majority of studies, whereas ED visits have remained relatively consistent. The study of psychosocial and direct effects has been notably underrepresented across all CMDs. Additionally, there is a paucity of research examining SRDs and burnout.

The initially elevated values stabilized as the pandemic progressed, which may suggest a successful adaptation to the novel social conditions. It thus appears prudent to provide assistance to susceptible patients with CMDs, particularly during the initial stages of a pandemic. Such a setting could take the form of an early temporary inpatient facility, for example, where patients with a score of high risk or high vulnerability in the vulnerability–risk matrix are shielded from social panic and hoarding. An alternative approach could be the provision of telemedical counseling or pandemic consultations. Moreover, training and enhancing resilience may prove to be a pivotal strategy for individuals with CMDs in the event of future pandemics. This approach could also be extended to susceptible segments of the general population. During periods of relative stability, individuals could be encouraged to engage in resilience-building strategies in collaboration with healthcare professionals. This would enhance their capacity for personal responsibility and resilience in the face of future crises.

In consideration of the potential ramifications of long-COVID cases, individual psychological counseling sessions following the recovery from infection may prove an efficacious strategy for the prevention of the documented elevated risk of developing an anxiety disorder. Thus, early counseling sessions could serve to mitigate initial uncertainty and facilitate a more positive outlook.

The existing literature on the subject presents only a limited number of studies on stress-related disorders, no studies on burnout, and no data on post-pandemic conditions. These are essential for a comprehensive assessment of long-term effects. Future research should take the form of observational studies, examining diagnosed CMDs in at least one time point before, during, and after the pandemic. This should consider both prevention measures and infection numbers in each country. This will enable the differentiation of short-term effects from chronic effects and the examination of stress-related disorders and burnout in relation to the consequences and impact of the COIVD-19 pandemic. In sum, the current body of literature is insufficient to provide meaningful recommendations for political and societal decision-makers; it can only provide preliminary indications. It is imperative that long-term data be collected beyond the conclusion of the pandemic.

## Figures and Tables

**Table 1:** Search string for the selected terms of the research question.

COVID	COVID-19 Pandemic OR SARS-CoV-2 OR 2019 nCoV OR Coronavirus Pandemic or “Severe Acute Respiratory Syndrome Coronavirus 2”
AND	
CMD	“Common mental disorder” OR Psychiatr* OR Anxiet* OR Phobi* OR “Anxiety disorder” OR “Phobic disorder” OR “Phobic anxiety disorder” OR “Panic disorder” OR “Generalized Anxiety disorder” OR “Generalised Anxiety disorder” OR “Obsessive-compulsive disorder” OR “Obsessive compulsive disorder” OR “Acute stress reaction” OR “Post-traumatic stress disorder” OR “Post traumatic stress disorder” OR “Posttraumatic Stress disorder” OR “Adjustment disorder” OR “Burn-out” OR “Burn out” OR “Burnout”
AND	
Adults	Adult
NOT	
Children/adolescents	Child* OR Adolescen*

The specific search codes were constructed individually for PubMed, Web of Science, and PsycInfo (Supplementary Materials).

**Table 2:** PECOS scheme of the inclusion and exclusion criteria for the search of effects of the COVID-19 pandemic on CMDs.

	Inclusion	Exclusion
**Participants/population**	Individuals aged 18 years or older; Patients with incidental or pre-existing mental disorders or new mental comorbidities, with the publications describing that the participants had been diagnosed with the respective common mental illness before or during the COVID-19 pandemic; Based on the criteria in the International Statistical Classification of Diseases and Related Health Problems (ICD-10), we consider the following common mental disorders and their subgroups, if they are clearly evident from the data: - Phobic disorders (agoraphobia with/without panic disorder, social phobia, and specific phobia); - Anxiety disorders (panic disorder and generalized anxiety disorder); - Obsessive-compulsive disorder (obsessional thoughts or ruminations and compulsive acts); - Reaction to severe stress (acute stress reaction, post-traumatic stress disorder, and adjustment disorder); - Problems related to life-management difficulty (burnout).	Other patient populations, children and adolescents.
**Exposures**	Exposure to the COVID-19 pandemic (i.e., study period includes data from before (pre), during (peri), and/or after (post) the COVID-19 pandemic); According to the WHO, the pandemic was proclaimed to begin on 11 March 2020 and to end on 5 May 2023.	Studies unrelated to the COVID-19 pandemic, studies only including pre-pandemic data, and exposure to other epidemic or pandemic infectious disease outbreaks (e.g., SARS, MERS, Ebola, HIV, influenza, or norovirus)
**Control/Comparators**	Comparison with other group(s) concerning the impact of the pandemic (e.g., severe COVID-19 infections in SMIs vs. CMDs); Suited longitudinal within-group peri-pandemic comparison in individuals with CMDs (e.g., progression of mental illness symptoms related to lockdown or changes in physician visits during the course of the pandemic).	Exposure to/assessment during any other pandemics, epidemics, or other macro-stressors (e.g., natural disasters).
**Outcomes**	Outcomes need to be questionnaire/self-report data provided by the individuals with pre-existing CMDs or by third persons (interviewer); patient, hospital, or other records (e.g., patient register data) that can fall into the psychosocial, ITE, or DTE categories, as further defined in Ascone et al. [8], are included: - PSE1: social environment; - PSE2: psychological status/coping; - PSE3: physiological status/health; - PSE4: lifestyle changes; - PSE5: traumatic experiences; - PSE6: job/occupation; - PSE7: environmental exposures; - PSE8: socio-economic factors; - PSE9: structural factors; - PSE10: overall quality of life. ITEs: Indirect total effects that relate to CMD; DTEs: Direct total effects that relate to CMD; Should further outcomes be identified that are not yet included in the above list, these will be added to the matrix scheme during the screening process, i.e., the matrix structure may be revised based on identified additionally relevant matrix outcome domains, e.g., quality of life.	Outcomes not capturing psychosocial, physical health, or COVID-19-related outcomes or mental illness-related effects.
**Study types**	Quantitative longitudinal survey studies; Quantitative repeated cross-sectional survey studies; Quantitative cross-sectional or retrospective survey studies; Longitudinal observational studies; Other types of empirical studies, i.e., qualitative with a coding scheme in any form (with/without control groups).	Preprints; Study protocols; Intervention studies; Treatment studies; Theoretical/discussion papers; Expert opinions or letters to the editor (if not containing any original data); Letters to the editor/editorials/commentaries.

**Table 3:** Evidence mapping of the number and frequency of study types and the identified PSEs, ITEs, and DTEs in the specified CMDs.

Matrix outcome dimension	PSE1: social environment, e.g., loneliness, conflicts, violence in partnerships/family, mobbing, lack of social (peer) support, lack of integration, discrimination	PSE2: psychological status/coping, e.g., poor self-confidence, neuroticism, deficits in emotional self-regulation, unfavorable family history, excessive demands, insecurity, destabilisa-tion, breakdown	PSE3: physio-logical status/health, e.g., reduced fitness, disturbed metabolism, abnormal inflammatory parameters, reduced heart rate variability, disabilities, allergy, susceptibility to infection	PSE4: lifestyle changes, e.g., poor nutrition, consumption of harmful substances, insufficient physical exercise, drug use, risk behavior, low mental activity, increased media consumption	PSE5: traumatic experiences incl. COVID-19-related regulations, e.g., previous life events, life-threatening experiences, witnessing severe COVID-19 events/lock-downs/isolation/incriminating reports/images	PSE6: job-/occupation-/related effects, e.g., work-related overload/underload, insecure job status, high-risk jobs (emergency medicine, military)	PSE7: environ-mental exposures, e.g., chemical, physical/biological pollution (lead, arsenic, pesticides, particulate matter), lack of exposure to nature, high building density, social density	PSE8: socio-economic effects, e.g., educational discrepancy/literacy gap, infodemic, disinforma-tion, poverty, language barriers, unresolved residence status, conspiracy theory, changed social climate	PSE9: structural effects, e.g., diminished access to healthcare/mental health services, disruptions in treatment continuity, reduced access to infrastructure/participation in social life	PSE10: overall quality of life	ITE: indirect total effects relating to CMD, e.g., higher incidence, change in symptoms/prognosis/relapse, new mental comorbidities, increased hospitalize-tion due to CMD, ED visits, suicides	DTE: direct virus-related effects, e.g., virulence of variants, higher contamination/mortality, interaction effects with psychotropic medication, increased hospitalize-tion, ventilation/lung machine/ECMO, Long-/Post-COVID
ratios and type of study designs *	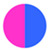	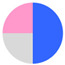	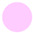	No evidence identified	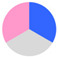	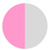	No evidence identified	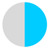	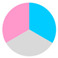	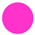	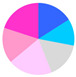	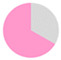
**N** studies on CMD	2	4	1	0	3	2	0	2	3	1	25	3
Country	US	CA, US	DE	-	CA, US	CA, US	-	CA, US, GB	CA, US, GB	DK	CA, US, DE, IT, GB, RS, DK, AU, NO, CZ, SE, ES, QA, NL	NO, US
**N** study size **	63 | 241	63|6.854	1.930.858	-	84 | 6.854	241 |6.854	-	6.854 | 14.210.507	241 | 14.210.507	201	36 | > 15 m.	241 | 10.463.672
**N** studies on specific CMD ***	AnxD: 0	AnxD: 0	AnxD: 0	AnxD: 0	AnxD: 0	AnxD: 0	AnxD: 0	**AnxD: 1**	**AnxD: 1**	AnxD: 0	**AnxD: 13**	**AnxD: 2**
	PAD: 0	**PAD: 2**	PAD: 0	PAD: 0	**PAD: 2**	**PAD: 1**	PAD: 0	**PAD: 1**	**PAD: 1**	PAD: 0	**PAD: 7**	PAD: 0
	PD: 0	**PD: 1**	**PD: 1**	PD: 0	**PD: 1**	**PD: 1**	PD: 0	**PD: 1**	**PD: 1**	PD: 0	**PD: 6**	PD: 0
	GAD: 0	**GAD: 1**	**GAD: 1**	GAD: 0	**GAD: 1**	**GAD: 1**	GAD: 0	**GAD: 1**	**GAD: 1**	GAD: 0	**GAD: 6**	GAD: 0
	**OCD: 2**	**OCD: 3**	OCD: 0	OCD: 0	**OCD: 2**	**OCD: 2**	OCD: 0	**OCD: 1**	**OCD: 2**	**OCD: 1**	**OCD: 9**	**OCD: 2**
	SRD: 0	SRD: 0	SRD: 0	SRD: 0	SRD: 0	SRD: 0	SRD: 0	SRD: 0	SRD: 0	SRD: 0	**SRD: 2**	SRD: 0
	ASR: 0	ASR: 0	ASR: 0	ASR: 0	ASR: 0	ASR: 0	ASR: 0	ASR: 0	**ASR: 1**	ASR: 0	ASR: 0	ASR: 0
	PTSD: 0	**PTSD: 1**	PTSD: 0	PTSD: 0	**PTSD: 1**	**PTSD: 1**	PTSD: 0	**PTSD: 1**	**PTSD: 1**	PTSD: 0	**PTSD: 5**	PTSD: 0
	AD: 0	AD: 0	AD: 0	AD: 0	AD: 0	AD: 0	AD: 0	AD: 0	AD: 0	AD: 0	**AD: 1**	AD: 0
	BU: 0	BU: 0	BU: 0	BU: 0	BU: 0	BU: 0	BU: 0	BU: 0	BU: 0	BU: 0	BU: 0	BU: 0

Note. * The larger the bubble plot, the more evidence; 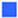 A = both longitudinal study and controlled by a suited control group (e.g., healthy control participants), 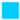 B = longitudinal study without control group within the same sample with CMD, 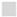 C = repeated cross-sectional study with control group comparison CMD vs. control group (peri- or post-pandemic), 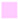 D = repeated cross-sectional study for different samples with CMD with at least two time points, 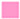 E = retrospective survey with control group, 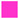 F = retrospective survey without control group; the color coding scheme is also available in the a priori protocol (DOI: 10.17605/OSF.IO/9EYD5). ** The first value is the number of the smallest study size (Paticipants pr health data), the second value is the number of the largest study size (participants or health data). *** AnxD = anxiety disorder (without subclassification/anxiety disorders with ICD code F.40-F.41), ASR = acute stress reaction, BU = Burnout, CMD = common mental disorders, GAD = generalized anxiety disorder, OCD = obsessive-compulsive disorder, PAD = phobic anxiety disorder, PD = panic disorder, PTSD = post-traumatic stress disorder, SRD = stress-related disorders (without subclassification/stress-related disorders with ICD code F.43). AU = Australia, CA = Canada, CZ = Czech Republic, DE = Germany, DK = Denmark, ES = Spain, GB = United Kingdom, IT = Italy, NL = Netherlands, NO = Norway, QA = Qatar, RS = Serbia, SE = Sweden, US = United States of America.

## Data Availability

The data that support the findings of this study are available from the corresponding author upon reasonable request.

## Supplementary Materials

The following supporting information can be downloaded at: https://www.mdpi.com/article/10.3390/ijerph22040478/s1, Table S1: Defined CMD according to ICD-10 based on the a priori protocol [57]; Table S2: Full search terms for the data search of CMD during the COVID-19 pandemic on the determined databases (*n* = 5325); Table S3: Metadata table of included studies [27,28,29,30,31,32,33,34,35,36,37,38,39,40,41,42,43,44,45,46,47,48,49,50,51,52]; Table S4: Exemplary vulnerability and risk exposure matrix of pandemic collective damage [8].
