# Supplementary material for: Mental Health in the Time of the COVID-19 Pandemic: A Scoping Review of Collateral Effects on Common Mental Disorders (CMDs)
Source: Int J Environ Res Public Health. 2025 Mar 23;22(4):478. doi: 10.3390/ijerph22040478 (PMC12027321; doi:10.3390/ijerph22040478)
Supplement: Supplementary file 1 [file ijerph-22-00478-s001.zip › ijerph-3492329-supplementary 3.20_revised 25.03.25.pdf]

Supplements

Table S1. Defined CMD according to ICD-10 based on the a priori protocol [57]

**F40.-**	**Phobic anxiety disorders**
**F41.-**	**Other anxiety disorders**
F41.0	Panic disorder [episodic paroxysmal anxiety]
F41.1	Generalized anxiety disorder
**F42.-**	**Obsessive-compulsive disorder**
**F43.-**	**Reaction to severe stress, and adjustment disorders**
F43.0	Acute stress reaction
F43.1	Post-traumatic stress disorder
F43.2	Adjustment disorders
**Z73.-**	**Problems related to life-management difficulty**
Z73.0	Burn-out

Table S2. Full search terms for the data search of CMD during the COVID-19 pandemic on the determined databases (*n*=5325)

**Pubmed (9th April 2024)**	
Filter	*Article language:* English, German
Results	2.348
String	(COVID-19 pandemic[tiab] OR SARS-CoV-2[tiab] OR 2019 nCoV[tiab] OR Coronavirus pandemic[tiab] or "severe acute respiratory syndrome coronavirus 2"[tiab]) AND ("Common mental disorder"[tiab] OR Psychiatr*[tiab] OR Anxiet*[tiab] OR Phobi*[tiab] OR "Anxiety disorder"[tiab] OR "Phobic disorder"[tiab] OR "Phobic anxiety disorder"[tiab] OR "Panic disorder"[tiab] OR "Generalized Anxiety disorder"[tiab] OR "Generalised Anxiety disorder"[tiab] OR "Obsessive-compulsive disorder"[tiab] OR "Obsessive compulsive disorder"[tiab] OR "Acute stress reaction"[tiab] OR "Post-traumatic stress disorder"[tiab] OR "Post traumatic stress disorder"[tiab] OR "Posttraumatic Stress disorder"[tiab] OR "Adjustment disorder"[tiab] OR "Burn-out"[tiab] OR "Burn out"[tiab] OR "Burnout"[tiab]) AND Adult*[tiab] NOT (Child*[tiab] OR Adolescen*[tiab])
**Web of Science (9th April 2024)**	
Filter	*All fields:* topic<br>*Languages:* English, German<br>*Categories:* psychiatry, public environmental occupational health, psychology multidisciplinary, psychology clinical, psychology, psychology social, psychology applied, psychology developmental
Results	2.168
String	(COVID-19 pandemic OR SARS-CoV-2 OR 2019 nCoV OR Coronavirus pandemic or "severe acute respiratory syndrome coronavirus 2") AND ("Common mental disorder" OR Psychiatr* OR Anxiet* OR Phobi* OR "Anxiety disorder" OR "Phobic disorder" OR "Phobic anxiety disorder" OR "Panic disorder" OR "Generalized Anxiety disorder" OR "Generalised Anxiety disorder" OR "Obsessive-compulsive disorder" OR "Obsessive compulsive disorder" OR "Acute stress reaction" OR "Post-traumatic stress disorder" OR "Post traumatic stress disorder" OR "Posttraumatic Stress disorder" OR "Adjustment disorder" OR "Burn-out" OR "Burn out" OR "Burnout") AND Adult* NOT (Child* OR Adolescen*)
**APA PsycInfo via EBSCOhost (9th April 2024)**	
Filter	*Document type:* journal article<br>*Language:* English
Results	809
String	TI ((COVID-19 pandemic OR SARS-CoV-2 OR 2019 nCoV OR Coronavirus pandemic or "severe acute respiratory syndrome coronavirus 2") AND ("Common mental disorder" OR Psychiatr* OR Anxiet* OR Phobi* OR "Anxiety disorder" OR "Phobic disorder" OR "Phobic anxiety disorder" OR "Panic disorder" OR "Generalized Anxiety disorder" OR "Generalised Anxiety disorder" OR "Obsessive-compulsive disorder" OR "Obsessive compulsive disorder" OR "Acute stress reaction" OR "Post-traumatic stress disorder" OR "Post traumatic stress disorder" OR "Posttraumatic Stress disorder" OR "Adjustment disorder" OR "Burn-out" OR "Burn out" OR "Burnout") AND Adult* NOT (Child* OR Adolescen*)) OR AB ((COVID-19 pandemic OR SARS-CoV-2 OR 2019 nCoV OR Coronavirus pandemic or "severe acute respiratory syndrome coronavirus 2") AND ("Common mental disorder" OR Psychiatr* OR Anxiet* OR Phobi* OR "Anxiety disorder" OR "Phobic disorder" OR "Phobic anxiety disorder" OR "Panic disorder" OR "Generalized Anxiety disorder" OR "Generalised Anxiety disorder" OR "Obsessive-compulsive disorder" OR "Obsessive compulsive disorder" OR "Acute stress reaction" OR "Post-traumatic stress disorder" OR "Post traumatic stress disorder" OR "Posttraumatic Stress disorder" OR "Adjustment disorder" OR "Burn-out" OR "Burn out" OR "Burnout") AND Adult* NOT (Child* OR Adolescen*))

Table S3. Metadata table of included studies [27 ‒ 52]

Authors (year)	Study type	Country/area, city<br><br>Timepoint(s) concerning pandemic	Outcome measures<br><br>Assessment tools<br><br>Control for infection status, i.e., if effects potentially confounded by COVID-19 infection	Summary of main outcomes
Anderson et al. (2022) [43]	D	US<br>four timepoints: pre vs. post of Delta variant (July 2021+April 2021); post of Delta variant vs. pre-pandemic (July 2021+July 2019); post case peak vs.during case peak (February 2021+December 2021); post case peak vs. pre-pandemic (February 2021+February 2019)	ED visits for 10 mental disorders and all mental health-related visits, F.40, F.41 =Phobic, Panic, GAD and F.43 = PTSD, ASR, AdjD and F.42 = OCD<br>→ no control for infection status	"1) Before and after Delta and before and after case peak stable number of visits (+/-10%), with a negative peak during April/May 2020 (most likeley due to doubled numbers of death, business closures), slight increase with following decrease in stress-related disorders, overall stable between 2019 and 2021<br>2) Delta to pre-Delta: entire sample stable, Asians had moderate to high increase in visits (anxiety 18% and trauma-stress related disorders 12% during Delta vs. pre-Delta<br>3) Delta to pre-pandemic: decrease up to 30%<br>4) During peak to after peak: stable, but more visits after peak than during peak, American Indian/Alaska Natives (42%!) had high increase in visits (trauma-stress related disorders<br>5) after peak to pre-pandemic: decreased in anxiety and trauma-stress, moderate increase in OCD (10%) at men from 50-64 years old"
Asmundson et al. (2022) [29]	C	Canada and US<br>two timepoints: onset of pandemic at 21.03.-01.04.20 and the end of the third wave 24.03.-04.05.21	self-report clinical diagnosis of mood and anxiety disorders (panic disorder, generalized anxiety disorder, social anxiety disorder), OCD, PTSD, COVID Stress Scale (CSS), COVID Disability Scale (CDS), PHQ4 for anxiety used to measure changes in disorder vs. healthy control<br>→ no control for infection status	"CSS was highest at people with panic disorder, all disorders had moderate to high stress levels in contrast to healthy control at timepoint 1, the score decreased to a moderate level at timepoint 2 in all disorders and the healthy control, people with panic disorder felt most disabled in the CDS and more disabled (40-50%) than the healthy control (20%), the PHQ Anxiety decreased from timepoint 1 to 2 in all disorders Authors summarize it as an adjustment reaction with the most perceived stress in PD. GAD, SAD and PTSD had also higher scores then the others."
Bendau et al. (2021) [30]	A	Germany<br>four timepoints between 27.03.-15.06.2020	self-report clinical diagnosis, self-report assessment of worsening of symptoms<br>PHQ4, COVID-related fear<br>→no control for infection status	People with anxiety disorders, had high symptoms, agoraphobia had higher symptoms of COVID-related fear, people with GAD had high symptoms at the beginning of pandemic, but also a higher decrease of symptoms over time
Bodini et al. (2023) [45]	F	Italy<br>three timepoints 2019, 2020, 2021	ED visits for a.o. anxiety disorders with ICD-10<br>→ no control for infection status	ED vitis for anxiety disorders decreaed in 2020 and 2021 in comparison to 2019 about 25-30%, authors discuss: higher incidence, that is reported in public could be just mild anxiety disorders, that do not need therapeutical treatment; people with anxiety disorders have more fear of contagion with COVID, so they do not go to the ED, this avoidance could also have leaded to severe anxiety disorders =higher stress symptoms, reduced consultations can also be because of mor tele-health interventions for psychiatric patients
Carlton et all. (2022) [31]	A	US<br>two timepoints (01-03/2021 + 05/2021)	clinical diagnosis of SAD through ADIS-5, FIVE, RSQ, SAFE, DASS-21<br>→ no control for infection status	Fear of illness and virus evaluation higher than in control, social distancing higher, avoidance behavior, anxiety, stress doubled higher, also negative coping strategies (disengagement coping, stress involuntary disengagement, stress involuntary engagement), primary and secondary control coping nearly the same in patient group and in healthy group
Carr et al. (2021) [32]	B	UK<br>one timespan: 01/2019 - 09/2020	incidence assessment through primary care data of a.o. anxiety disorders<br>→no control for infection status	events of self harm relative stable from prepandemic to pandemic, prescription of benzodiazepines in age group of 80+ very high at the beginning of the first wave of pandemic (peak), incidence of anxiety disorders decreased in all age groups and then increased after the first wave, people in deprived areas had the strongest decrease in self harm and referral to mental health services, authors discuss the reason for it, was the reduced access to mental health services due to restrictions
Dennis et al. (2023) [36]	E	US<br>one timespan 15.04.-02.07.21	OCD symptoms (DOCS), DASS-21 anxiety, COVID-19 concern scale (CNS), impairment scale in school/work, social life, family<br>→ control for infection status: mild or severe COVID infection with/without hospitalization	COVID concern scale twice higher than the control group, 64% had worse symptoms since outbreak of pandemic, 62% say concerns about COVID increase disstress more than the OCD disorder, impairment by COVID is twice higher than the control group, anxiety symtoms in mean 5 times higher than in the control group, people with OCD had more lost of loved one by COVID than controls and were more hospitalized or had severe infection process than the controls, impairment scale (impact of pandemic on work, family, social life) was also higher than in the controls
Golubovic et al. (2022) [35]	E	Serbia<br>three timepoints: 05-08/2020 compared to same period in 2019 and 2018	number of clinical diagnosed a.o. adjustment disorder at inpatients, number of suicide behaviour<br>→ no control for infection status	Clinical diagnosis of adjustment disorder increased from 2018 (2%), to 2019 (5%), to 2020 (11%), after lockdown in Serbia there were 12% with suicide ideation and 32% with suicide attempt, authors discuss, that restrictions and fear of COVID led to reduced utilisation of mental health services in patients with mental disorders, increased mental pain and led to higher suicide rates, important are patients that have never been diagnosed with AD before pandemic, they developed AD due to COVID stress

Hezel et al. (2022) [39]	A	US<br>four timepoints during 04/2020 - 12/2020	clinical diagnosed by physicians via telephone<br>based MINI interview, assessment of symptoms with OCI-R, anxiety with DASS-21, resilience scale, positive changes in social life during pandemic, e.g. more time with family assessed with EPII<br>→ no control for infection status	"Patients with OCD had more anxiety symptoms, more<br>OCD symptoms, alittle bit less resilience than control, after beginning of pandemic one patient had no longer OCD, one patient had new comorbid (panic disorder), healthy control did not change in mental health during pandemic, but OCD patients had worsening of symtpoms (40%), process of symptom severity at OCD patients was high at beginning of pandemic, than lower (a.e. adaption), and then increased again to the state of the beginning, also with anxiety, OCD patients said they have positive changes in life, but less than the healthy controls, at patients with higher level of resilience (both in OCD and healthy) had more stable symptoms over time and people with less resilience had more instable symptoms, resilience could be a good treatment factor for future pandemic"
Højgaard et al. (2021) [41]	F	Denmark<br>one timespan: 06.04.-29.04.2020	patients from Danish OCD association, clinical diagnosed<br>Y-BOCS for symptom severity of OCD, quality of life questionnaire<br>→ no control for infection	Over 90% of patients reporting a worsening of symptoms or no change of symptoms since outbreak of pandemic, positive for obe third of OCD patients: obsessive thoughts are now more acceptable, onl 8% had treatment because of pandemic, 65% had contaminated-related symptoms already before pandemic, 15% reporting a massive reduction of quality of life associated with higher worsening of OCD symptoms, 50% reporting higher anxiety, for 61% pandemic lead to worsening of symptoms, especially in women, in patients with comorbidity and contamination symptoms, 10% reportes an increase of symptoms, because symptoms are more accepted and people were at home = lower anxiety of being contaminated, three quarters reported at least little bit reduction of quality of life
Jacob et al. (2021) [40]	D	Germany<br>two timepoints during 01-06/2020	number of clinical diagnosed anxiety disorders with F.41 spectrum, at least one consultation, number of prescription of psychotropic medication (anxiolytics etc.)<br>→ no control for infection status	At beginning of pandemic in 03/2020 34% more anxiety disorder praevalence (prior disorder and new disorder is unclear) until 06/2020 19% more in comparison to 2019,newly diagnosed (=incidence, no disorder prior) anxiety disorders in 03/2020 40%+ until 06/2020 28%+, the praevalence of psychotropic medications against anxiety disorder (antidepressants, anxiolytics, herbal sedativa) decreased from 2020 to 2019, but there were more physical comorbidites (Asthma and COPD) in people diagnosed with anxiety disorder, people were also older with new diagnosis ((51-60 years old), incidence risk of developing an anxiety disorder is probably higher in people with an increased risk in severe COVID-infections due to pulmonary diseases. authors discuss, results could be underestimated, because the consultations were just at general practices not in psychiatric hospitals, maybe also overestimated, because people just had "normal" anxiety symptoms as part of an adaption process during pandemic
Jagadheesan et al. (2022) [34]	E	Australia<br>two timepoints: 16.03.-12.05.20 + 16.03.-12.05.19	demographic data, clinical diagnosis of any anxiety disorders, not clear, if it is an pre-existing diagnosis or new diagnosis (prevalence assessment)<br>→ no control for infection	Visits due to anxiety disorders increased from 15 to 20% during the 9 weeks of lockdown, at the end of lockdown number decreased again, other results not evaluable, because mixed with other disorders, authors discuss reason for increased anxiety disorders (especially at ageing of 26-45), because of reduced access to facilities, financial hardships and other fears, that require a face-to-face treatment in ED
Jensen et al. (2023) [27]	E	Norwegian<br>two timepoints: 2017-2019 and 2019-2021	diagnosis through ICPC-2 codes for coding primary care diagnosis of anxiety disorders, phobia, PTSD<br>→ no control for infection status	"Mean difference of visits due to anxiety disorders were 27% during pandemic, in all age groups were peaks at beginning of strict lockdowns/social distancing, after that, they decreased again<br>mean difference of visits due to PTSD were even more higher with 37% difference between expected and real visits during pandemic, especially at beginning of lockdowns and in younger patients<br>significant difference in OCD and phobia during spring of 2020 and 2021 in older people (40-65), otherwise no strong difference, authors discuss reason for this because of reduced access to medical health system, this could have worsened symptoms, but also fear of infection in public"
Kasal et al. (2023) [33]	C	Czech<br>three timepoints: 11/2017 + 05/2020 + 11/2020	interviews with MINI for diagnosis, suicide risk, suicide behaviour, suicidal thoughts in anxiety disorders (panic disorder, generalized anxiety disorder, agoraphobia, social phobia, posttraumatic stress disorder)<br>→ no control for infection status	"Praevalence change of panic disorder: stable, social phobia doubled from 2 to 4%, agoraphobia from 5 to 9%, GAD from 3 to 6%, PTSD from 1 to 3%, CAVE: there are both patient groups within, the pre-existing and the newly diagnosed<br>suicide risk (SR) in patients with pre-existing anxiety disorders and PTSD is 13% higher in May 2020 and 21% higher in Nov. 2020 (healthy population: 4% and 6%), so both have an increased risk, but pre-existing disorders are more at risk, suicidal thoughts and behavior in healthy population during pandemic 5% higher, in people with pre-existing disorders more than twice higher, at beginning of pandemic 11% and in 11/2020 20% in anxiety disorders and PTSD"
Moreno-Martos et al. (2024) [49]	A	Norway and Sweden<br>one timespan: 01.01.2018 - 31.12.2020	change of prescripted psychotropic medication in clinical diagnosed anxiety disorders with ICD code, hospitalization due to mental disorder, outpatient visits, data from national registries<br>→ no control for infection status	Anxiety disorders: praevalence in Norway 5%, Sweden 4%, anxiolytic medication reduced at beginning of pandemic in 03/2020 in comparison to 2019, then increased a little bit again until the end of 2020, hospitalization due to anxiety disorders: -33% in Norway, stable in Sweden, outpatient visits in Norway reduced than increased, in Sweden stable, authors discuss, that praevalence is not incidence and not every change in symptoms result in a need for treatment
Nemani et al. (2021) [37]	E	US, New York City<br>one timespan: 3.3.-31.5.2020	mortality in patient group with anxiety disorders with ICD-10 F.40 + F.41<br>→ control for infection status	Anxiety disorders have no higher mortality risk at the beginning of pandemic
Pfoh et al. (2021) [44]	F	US<br>three timepoints during 1.8.2019 - 31.10.2020	clinical diagnosis in visits with ICD-10 F.41 + F.43<br>→ no control for infection status	Increase in initial period 04/20-07/20 10% vs. 9% pre-period and stayed stable high until end of 2020

Raventós et al. (2022) [46]	B	Spain<br>three timepoints: 01.03.2018 - 31.03.2021, pre-lockdown, lockdown, post lockdown	clinical new diagnosis of F.41<br>→ no control for infection status	Increase of anxiety disorders, incidence rate (IR) per 100.000 pre lockdown 151, lockdown 183 (peak), post lockdown 162, but lower than expected, increase of 37% in first lockdown
Sacco et al. (2022) [42]	E	US, New York City<br>two timespans: 15.03.-31.07.20 vs. same period in 2017,2018,2019	ED visits due to clinical diagnosis of F.41<br>→ no control for infection status	ED visits at the beginning of pandemic increased compared to 2017+2018+2019, but decreased again, result: relative stable numbers of visits, just at the beginning, RR 1,4 times higher to be diagnosed with anxiety disorder during first half year of pandemic than prepandemic, authors discuss no comparison postpandemic
Siddiqui et al. (2022) [52]	C	Qatar<br>two timepoints: before and during pandemic (no exact timeline)	clinical diagnosis of pre-existing OCD with fear of contamination and washing compulsion as major symptoms, symptom severity with Yale-Brown obsessive compulsive scale<br>→ no control for infection status	Significant results in patients with duration of <10 years of disorder had an increase in symptoms during pandemic, Cave: unclear what kind of timespan the data was collected
Storch et al. (2021) [51]	F	US<br>three timespans during pretreatment vs. just before beginning of pandemic vs. 17.07.-04.08.2020 (during pandemic)	clinical diagnosis of OCD of clinicans (they filled out the survey) with GOCS<br>→ no control for infection status	High symptoms prior treatment, lower just before pandemic, increasing again since beginning of pandemic (Cave: assessment of clinicans, not patients)
Teixeira et al. (2022) [38]	C	US<br>three timepoints: 2018, 2019, 2020	clinical diagnosis of anxiety and OCD with F.40+F.41+F.42<br>→ control for infection status: comparison group, patients with and without COVID und severity of infection process (ventilation etc.)	No change or no significant change in Incidence rates (IR) before and during pandemic in anxiety disorders and OCD, but significant difference in infection status, IR rates round about one third higher (28 vs. 47%) for anxiety disorders and a little bit higher in OCD (0,4 vs. 0,6%), OR btw. 1,1 and 3,0! for positive correlation of anxiety and severe COVID infection (1-9 = mild to severe), no higher OR for OCD and COVID severity, that means people with infection experience had higher risk for developing an anxiety disorder, higher the severity, higher the risk, authors discuss the stable number of anxiety disorders with higher avoidant behaviors, so that people suffer more, but they don't look for medical help, they also discuss reasons for correlation btw. infection severity and OR for anxiety disorder in 1. biological responses as inflammatory processes due to infection, hypoxy and 2. psychological reactions as traumatic experiences in hospital treatment with ventilation
Ten Have et al. (2023) [28]	B	Dutch<br>one timespan: 11/2019 - 03/2022	clinical interview with CIDI 3 of a.o. anxiety disorders include panic disorder, agoraphobia, social anxiety disorder or social phobia, specific phobia, generalized anxiety disorder<br>→ no control for infection status	Praevalence rates before and during pandemic did not differ significantly with 15%, over time since 2007 until 2022 number of anxiety disorders increased from 10 to 16%, but not during pandemic
White et al. (2023) [50]	F	US<br>two timespans: 1.3.2019 -28.02.2020 (pre COVID) and 01.03.2020-28.02.2021 (during COVID)	inpatients with diagnosed anxiety disorders in a psychiatric hospital<br>→ no control for infection status	Use of medication against anxiety decreased during pandemic in comparison to prepandemic, authors discuss the frame of a protecting hospital environment could be helpful to anxiety disorders during pandemic, because inpatients are not so confrontated with the behavior of society (panic, hoarding purchases)
Winkler et al. (2020) [47]	D	Czech<br>two timespans: 2017 + 06.-20.05.2020	MINI interview web-based, anxiety disorders (panic disorder, GAD, agoraphobia, social anxiety disorder, PTSD)<br>→ control for infection status in anxiety disorders, but either if tested positive or negative	Anxiety disorders in whole increased from 2017 to May 2020 from 8 to 13%, Panic from 0 to 1%, GAD from 3 to 5%, Agora from 5 to 8%, Social from 2 to 3%, PTSD from 1 to 2%, COVID health related worries and economic worries are more risk (OR 1,7 and 1,4) for developing an anxiety disorder
Winkler et al. (2021) [48]	D	as above + 11/2020 (second wave)	procedure as in the publication above	Results are extended by praevalence in the second wave in 11/2020: more risk at younger female adults (18-25), and people with less economic status, Anxiety disorders in whole from 2017 to May 2020 to Nov. 2020 from 8 to 12 to 13%, Panic 0,2,0,8,1,1; GAD 3,5,5; Agora 5,8,9; Social 2,3,3; PTSD 1,2,2

Table S4. Exemplary vulnerability and risk exposure matrix of pandemic collective damage [8]

Vulnerability and risk exposure matrix of pandemic collective damage	Vulnerability by sex or gender		Vulnerability by age				Vulnerability due to difficult accessibility or minority status				Vulnerability according to specific health conditions							Vulnerability through care	Vulnerability due to work status	
											Mental disorders		Somatic disease or somatic status							
	Women	Men	Children	Adoles-cents	Middle age group	Elderly people	Homeless people	Migrants	Refugees	Ethnic minorities	Common mental disorders	Severe mental illnesses	Chronic diseases	Infections	Disabilities	COVID-19 survivors	Pregnancy	Carers (e.g. parents of underage children, family carers)	Healthcare staff (e.g. staff in intensive care units)	Carers of the seriously ill (e.g. street workers)
**Pre-existing risk factors***																				
Mental health burden and vulnerability	1	1	2	2	1	2	3	1	3	2	2	3	2	1	2	0	1	1	2	2
Somatic health burden and vulnerability	0	0	0	0	0	3	2	0	2	1	1	3	3	3	2	0	1	1	1	1
Low income	0	0	0	0	0	1	3	1	3	1	1	3	1	0	1	0	0	1	1	1
Critical stage of development	0	0	2	3	0	2	0	1	2	1	0	2	0	0	1	0	2	1	0	0
Low level of education	0	0	0	0	0	0	2	1	2	1	0	2	0	0	1	0	0	0	0	0
Restricted access to healthcare	0	0	1	1	0	1	3	1	2	2	0	3	1	1	1	0	0	0	0	0
Stressful / insecure work or unemployment	0	0	0	0	1	0	2	1	3	1	1	2	0	2	2	0	1	1	3	3
Small (local) social support network	0	0	0	0	0	1	3	1	2	1	0	3	1	0	1	0	0	0	0	0
Minority status	0	0	0	0	0	0	3	2	3	3	0	2	1	0	2	0	0	0	0	0
Impaired physical abilities	0	0	0	0	0	2	2	1	2	1	1	2	3	2	2	0	1	0	0	0
Impaired mental abilities or skills (incl. lack of language skills)	0	0	0	1	0	1	3	2	3	2	1	3	2	2	2	0	0	0	0	0
High dependence on external help	0	0	2	1	0	1	2	1	3	1	1	2	3	2	2	0	0	0	0	0
Limited accessibility (i.e. difficult to reach)	0	0	0	0	0	1	3	1	3	2	0	3	0	0	1	0	0	0	0	0
**Vulnerability risk value**	**1**	**1**	**7**	**8**	**2**	**15**	**31**	**14**	**33**	**19**	**8**	**33**	**17**	**13**	**20**	**0**	**6**	**5**	**7**	**7**
**Pandemic-related risk factors***																				
Risk of exposure to the virus	0	0	2	2	1	2	3	2	3	3	0	2	3	2	1	0	0	1	3	3
Death of a parent, carer or close relative	0	0	0	0	1	2	1	2	3	3	0	2	1	0	0	0	0	3	1	1
Direct contact with active cases	0	0	2	2	1	2	3	2	3	3	0	3	2	0	1	3	0	2	3	3
Restricted access to healthcare	0	0	0	0	0	1	2	2	3	2	1	2	1	0	2	0	0	0	0	0
Prolonged isolation	0	0	1	1	1	2	1	2	2	2	1	3	3	2	2	3	1	1	1	1
Stress at work	1	1	0	1	2	0	0	2	1	1	1	1	0	1	1	0	1	2	3	3
Burn-out	1	1	0	0	1	1	0	1	1	0	1	0	0	0	0	0	0	2	3	3
Stigmatisation	0	0	0	1	0	1	2	2	3	1	1	1	1	0	1	2	0	0	1	1
Social rejection	0	0	0	0	0	1	3	2	2	1	1	1	1	0	1	2	0	0	1	1
Misinformation	0	0	2	2	1	1	3	3	3	3	0	3	0	0	2	0	0	0	0	0
Loss of support from peers (e.g. closure of school or workplace, place of residence)	0	0	2	3	1	3	1	1	1	1	0	1	1	2	1	0	1	2	1	1
Academic loss	1	1	1	2	0	0	0	1	0	0	1	0	0	0	0	1	1	0	0	0
Uncertainty (uncertainty as a psychological state)	0	0	1	2	1	1	1	2	1	1	1	2	2	3	1	3	2	2	3	2
**Vulnerability risk value in the pandemic**	**3**	**3**	**11**	**16**	**10**	**17**	**20**	**24**	**26**	**21**	**8**	**21**	**11**	**10**	**13**	**14**	**6**	**15**	**16**	**15**
**Total risk value (expected collateral damage)**	4	4	18	24	12	32	51	38	59	40	16	54	28	23	33	14	12	20	23	22

* Various combinations (factors may simply not apply or may not be specific to this group) and manifestations of these factors characterise the vulnerable group, which has an increased potential vulnerability to collateral damage (due to these hazards). A distinction can be made between generic and specific factors, whereby the generic factors allow a certain degree of comparability between the groups (common vulnerabilities) and the specific factors are unique to the respective group.

** Rating: 0 = no vulnerability, no risk; 1= low vulnerability, low risk; 2= medium vulnerability, medium risk; 3 = high vulnerability, high risk
